# Identification of potential organ donors after aneurysmal subarachnoid hemorrhage in a population-based neurointensive care in Eastern Finland

**DOI:** 10.1007/s00701-018-3600-2

**Published:** 2018-06-27

**Authors:** Olli-Pekka Kämäräinen, Jukka Huttunen, Antti Lindgren, Maarit Lång, Stepani Bendel, Ari Uusaro, Ilkka Parviainen, Timo Koivisto, Helena Isoniemi, Juha E. Jääskeläinen

**Affiliations:** 1 Neurosurgery of KUH NeuroCenter, Kuopio University Hospital, PB 1777, 70211 Kuopio, Finland; 2 Institute of Clinical Medicine, School of Medicine, Faculty of Health Sciences, University of Eastern Finland, Kuopio, Finland; 3 Neurointensive Care, Kuopio University Hospital, Kuopio, Finland; 4 Transplantation and Liver Surgery Clinic, Abdominal Center, Helsinki University Hospital, Helsinki, Finland

**Keywords:** Brain death, Intensive care, Intracranial aneurysm, Organ donation, Potential organ donor, Subarachnoid hemorrhage

## Abstract

**Background:**

To analyze the organ donation action in population-based neurointensive care of acute aneurysmal subarachnoid hemorrhage (aSAH) and to seek factors that would improve the identification of potential organ donors (PODs) and increase the donor conversion rate (DCR) after aSAH.

**Methods:**

The Kuopio Intracranial Aneurysm Database, prospective since 1995, includes all aSAH patients admitted to the Kuopio University Hospital (KUH) from its defined Eastern Finnish catchment population. We analyzed 769 consecutive acute aSAH patients from 2005 to 2015, including their data from the Finnish Transplantation Unit and the national clinical registries. We analyzed PODs vs. actual donors among the 145 (19%) aSAH patients who died within 14 days of admission. Finland had implemented the national presumed consent (opt-out) within the study period in the end of 2010.

**Results:**

We retrospectively identified 83 (57%) PODs while only 49 (34%) had become actual donors (total DCR 59%); the causes for non-donorship were 15/34 (44%) refusals of consent, 18/34 (53%) medical contraindications for donation, and 1/34 (3%) failure of recognition. In 2005–2010, there were 11 refusals by near relatives with DCR 52% (29/56) and only three in 2011–2015 with DCR 74% (20/27). Severe condition on admission (Hunt and Hess grade IV or V) independently associated with the eventual POD status.

**Conclusions:**

Nearly 20% of all aSAH patients acutely admitted to neurointensive care from a defined catchment population died within 14 days, almost half from cardiopulmonary causes at a median age of 69 years. Of all aSAH patients, 11% were considered as potential organ donors (PODs). Donor conversion rate (DCR) was increased from 52 to 74% after the national presumed consent (opt-out). Implicitly, DCR among aSAH patients could be increased by admitting them to the intensive care regardless of dismal prognosis for the survival, along a dedicated organ donation program for the catchment population.

## Introduction

The shortage of donated organs and the high mortality in the waiting lists for transplantation are a worldwide challenge, also in all European countries [7, 19]. Leading causes of brain death during intensive care include aneurysmal subarachnoid hemorrhage (aSAH) [6, 18], spontaneous intracerebral hemorrhage (ICH), and traumatic brain injury (TBI) [7, 12, 17, 20]. In a Canadian population-based cohort (1994–2011), only 1930 (2.2%) of the 87,129 patients who died from acute brain catastrophe (TBI, SAH, ICH, others) became actual organ donors; SAH led two times more often to organ donation than TBI [17]. In a Southern Finnish cohort of 955 acute ICH patients (2005–2010), 254 (27%) had died within 14 days, and only eight (3%) ended up as organ donors [20]. Improved donor conversion rates (DCRs) would alleviate the chronic shortage of legally transplantable organs [2]. In case of brain death during intensive care, main reasons for refraining from organ donation are reported to be (i) medical unsuitability and (ii) refusal of consent by near relatives [7, 12, 17]. In addition, acute care physicians may not admit patients with dismal prognosis for the survival after acute brain catastrophes to intensive care as potential organ donors (PODs).

In 2010, an international expert panel (neurology, neurotraumatology, intensive care medicine, transplantation medicine, intensive care ethics, and organ procurement management) suggested criteria for intensive care to identify a patient with a reasonable probability to become brain dead (imminent brain death) after acute brain catastrophe [3]. Two validated coma scales (Glasgow Coma Scale and the FOUR Score), brain stem reflexes, and respiration were used to define imminent brain death. The algorithm defined an actual pool of potential organ donors (PODs), with the exclusion of advanced age and medical contraindications.

In 2017 in Finland (population 5.5 million), 374 recipients received organs from a total of 116 donors, 29 of them live near-relative donors of kidneys (Finnish National Transplantation Registry). In the national waiting list, including 460 kidney patients in dialysis, 2–10% had died or became withdrawn, depending on the organ needed. To improve national DCR, the presumed consent (opt-out) was implemented in 2010. Organ donation after cardiac arrest has not been assigned in Finland.

To date, few studies have analyzed DCR in aSAH [7, 12, 15, 17]. The Kuopio Intracranial Aneurysm Patient and Family Database includes all aSAH patients admitted to the Kuopio University Hospital (KUH) from its defined Eastern Finnish catchment population [8]. In our previous study, the mortality of 1657 aSAH patients after acute hospitalization in1980–2007 was 11% at 3 days, 22% at 30 days, and 27% at 12 months [10]. In the present study, we analyzed 769 consecutive acute aSAH patients admitted to KUH Neurointensive Care from 2005 to 2015, including their data from the Finnish Transplantation Unit and the national clinical registries. We identified and analyzed PODs to seek factors that would (i) improve the identification of PODs and (ii) increase DCR after aSAH.

## Methods and materials

### Brain death and organ donor action in Finland

Finland implemented brain death in 1971 [11]. The national guidelines on organ donation and transplantation by the Ministry of Social Affairs and Health define two types of death: brain death and cardiac death. Brain death due to an acute brain catastrophe is diagnosed, after exclusion of confounding factors, by a board-certified neurosurgeon, neurologist, or pediatric neurologist, together with a board-certified anesthesiologist or intensivist. The diagnosis is based on the clinical examination, verification of the absence of brain stem reflexes and the absence of spontaneous breathing with arterial partial pressure of blood carbon dioxide ≥ 8 kPa [16]. The absence of brain arterial circulation can be verified by CT angiography or 4-vessel catheter angiography, but is not mandatory. In Finland, all organ removals and transplantations are performed and archived by the Transplantation Center of the Helsinki University Hospital (HUS). All donors and recipients are also registered in Scandiatransplant, the official organ exchange organization of Nordic Countries.

### Kuopio University Hospital (KUH) and aSAH management protocol in Eastern Finland

**Fig. 1:**
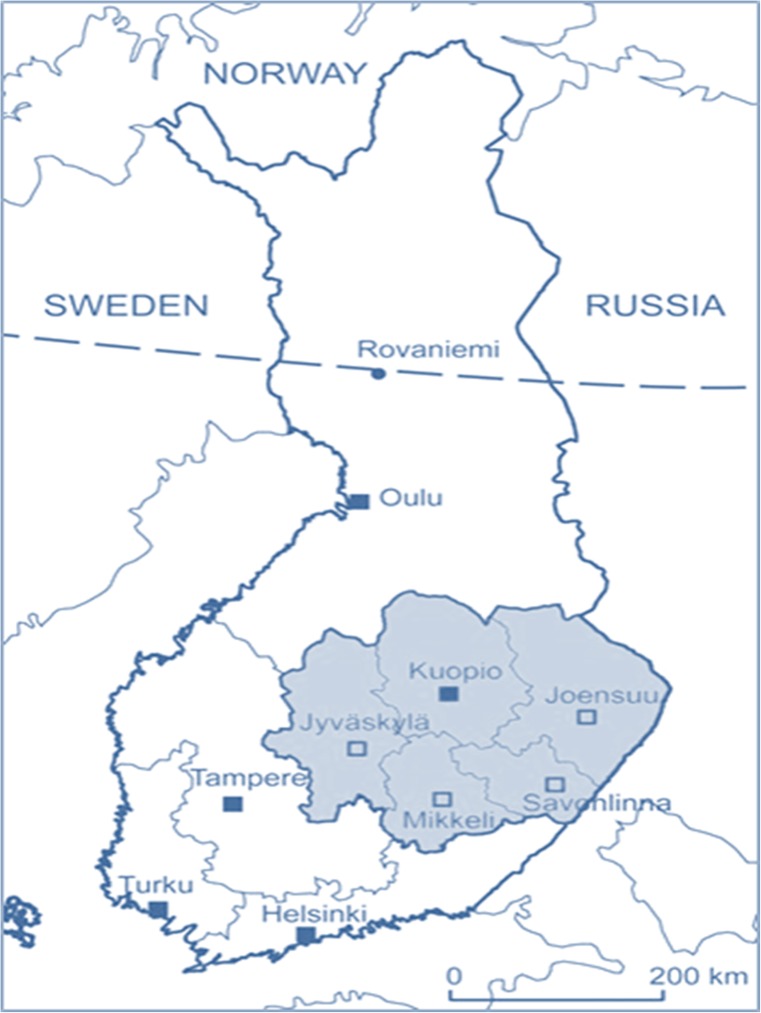
Map of the catchment area of the Kuopio University Hospital (KUH), containing 4 central hospitals (Joensuu, Jyväskylä, Mikkeli, Savonlinna) with acute neurology and CT services. All patients with acute aSAH are referred to KUH Neurosurgery and KUH Neurointensive Care

KUH, one of the five university hospitals in Finland, is an academic, non-profit, publicly funded tertiary center, serving a defined catchment population in Eastern Finland (Fig. 1). The KUH area contains four central hospitals with catchment areas of their own (Fig. 1), providing full-time neurology, intensive care, and CT services.

KUH Neurosurgery and KUH Neurointensive Care have exclusively provided full-time (7 days, 24 h) acute and elective neurosurgical services for the KUH catchment population [8–10]. All cases of SAH diagnosed by CT or spinal tap are acutely transferred to KUH for neurointensive care, neuroradiology (4-vessel catheter angiography and/or CT angiography), and neurosurgical treatment.

Neurointensive care is provided regardless of the condition on admission, including Hunt and Hess comatose grade V patients. A dedicated team of neurointensivists, neurosurgeons, and neuroradiologists coordinates the aSAH treatment. KUH Neurovascular group provides microsurgical or endovascular occlusion of the ruptured aneurysm; cases with significant ICH are immediately treated microsurgically. The protocol follows international recommendations in detail [5, 14, 21], aiming to prevent further brain damage due to re-bleeding, increased intracranial pressure (ICP), hydrocephalus, electrolyte disturbances, seizures, cardiac and pulmonary dysfunction, fever, hyperglycemia, and development of delayed brain ischemia. The protocol includes, when appropriate, e.g., external ventricular drainage (EVD), parenchymal ICP monitoring, endovascular procedures, and intra-arterial nimodipine infusion in case of delayed brain ischemia, as well as decompressive craniectomy (DC).

### Kuopio Intracranial Aneurysm Patient and Family Database

This database, prospective since 1995, contains all of the patients, including family history, with angiographically verified aSAH from the KUH catchment population. The phenotype and outcome of intracranial aneurysm disease in Eastern Finland have been analyzed in several studies [8–10]. The clinical data, using the Finnish personal codes, have been fused from the national registries, including prescribed medicines, hospital diagnoses, and causes of death (Table 1).

**Table 1:** Characteristics of the 769 consecutive aneurysmal subarachnoid hemorrhage (aSAH) patients acutely admitted to the Neurointensive Care of Kuopio University Hospital from its Eastern Finnish catchment population from 2005 to 2015. Characteristics of the 145 patients who died within 14 days after admission are presented according to their organ donor status

	All aSAH patients *n* = 769	aSAH patients dead within 14 days after admission to neurointensive care				
		All *n* = 145	Potential organ donors (PODs) in retrospect *n* = 83/145 (57%)			Cardiac death *n* = 62 (43%)
			All *n* = 83	Actual donors *n* = 49/83 (59%)	Non-donors *n* = 34/83 (41%)	
Median age (years)	55 (17–87)	60 (30–86)	56 (30–85)	49 (33–72)	63 (30–85)	69 (40–86)
Female	464 (60%)	85 (59%)	50 (60%)	28 (57%)	22 (65%)	35 (57%)
Hypertension Diabetes	253 (33%) 51 (7%)	62 (43%) 19 (13%)	29 (34%) 7 (8%)	14 (29%) 1 (2%)	14 (41%) 6 (18%)	34 (55%) 12 (19%)
Hunt and Hess grade						
I II III IV V	155 (20%) 211 (27%) 134 (18%) 171 (22%) 98 (13%)	6 (4%) 6 (4%) 19 (13%) 44 (30%) 70 (49%)	2 (2%) 3 (4%) 10 (12%) 18 (22%) 50 (60%)	2 (4%) 2 (4%) 6 (12%) 11 (22%) 28 (57%)	0 (0%) 1 (3%) 4 (12%) 7 (21%) 22 (65%)	4 (7%) 3 (5%) 9 (15%) 26 (42%) 20 (32%)
Location of aneurysm						
ICA ACA MCA BA PICA	176 (23%) 194 (25%) 227 (30%) 141 (18%) 31 (4%)	38 (26%) 38 (26%) 41 (28%) 20 (14%) 8 (6%)	21 (25%) 23 (28%) 24 (29%) 12 (14%) 3 (4%)	12 (25%) 13 (26%) 16 (33%) 7 (14%) 1 (2%)	9 (27%) 10 (29%) 8 (24%) 5 (15%) 2 (6%)	17 (27%) 15 (24%) 17 (27%) 8 (14%) 5 (8%)
Median aneurysm size	7 mm	8 mm	10 mm	10 mm	9.5 mm	7 mm
Multiple aneurysms	232 (30%)	34 (23%)	19 (23%)	9 (18%)	10 (29%)	15 (24%)
ICH	220 (29%)	68 (48%)	39 (47%)	23 (47%)	16 (47%)	30 (48%)
IVH	291 (38%)	100 (69%)	56 (68%)	32 (66%)	24 (71%)	44 (71%)
Acute hydrocephalus	364 (48%)	99 (68%)	49 (59%)	27 (55%)	20 (59%)	50 (81%)
Occlusive aneurysm therapy						
Microsurgical Endovascular None	24 (32%) 429 (56%) 98 (13%)	19 (13%) 43 (30%) 83 (57%)	8 (10%) 17 (20%) 58 (69%)	5 (10%) 12 (25%) 32 (65%)	3 (9%) 5 (14%) 26 (77%)	11 (18%) 26 (42%) 25 (40%)
Decompressive craniectomy	46 (6%)	14 (10%)	3 (4%)	3 (6%)	0	11 (18%)
Median time to death from admission (days)	N/A	3 (0–14)	1 (0–14)	1 (0–14)	1 (0–12)	8 (0–14)

*ICH* intracerebral hematoma, *IVH* intraventricular hematoma, *ACA* anterior cerebral artery, peripheral segments and anterior communicating artery, *BA* basilar artery trunk and bifurcation, *ICA* internal carotid artery trunk and bifurcation, posterior communicating artery, *MCA* middle cerebral artery and peripheral segments, *PICA* posterior inferior cerebellar artery

### Study population

**Fig. 2:**
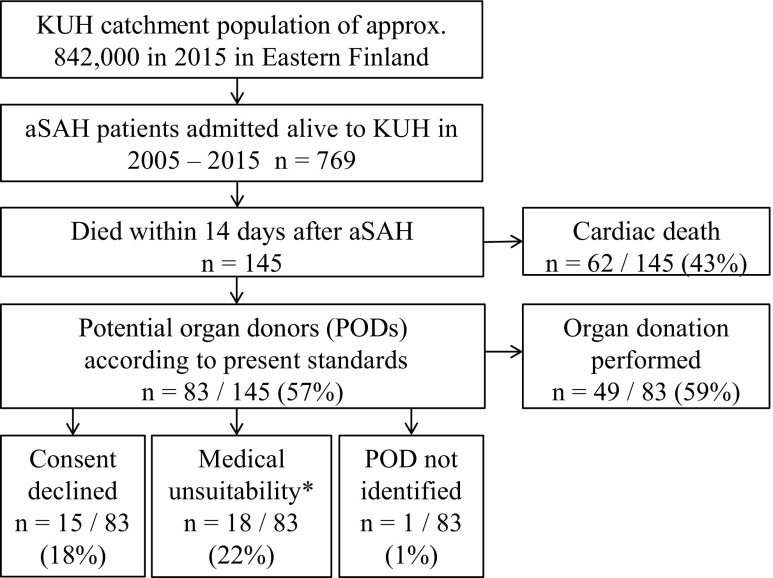
A total of 769 patients had been admitted acutely for aneurysmal subarachnoid hemorrhage (aSAH) to the neurosurgical and neurointensive care at the Kuopio University Hospital (KUH) between 2005 and 2015 from its defined Eastern Finnish catchment population. A total of 145 aSAH patients had died within 14 days, and 49 of them had become actual organ donors. In retrospect, we identified 83 potential organ donors (PODs) (Tables 1 and 2) and analyzed causes for the non-donorship among the remaining 34 PODs. * Defined medical unsuitability for organ donation: cardiovascular, pulmonary, diabetic and/or renal conditions; autoimmune disease; systemic infection; cardiac failure during donor management

The basic study population consisted of 769 consecutive, acutely admitted, angiographically verified aSAH patients to KUH Neurointensive Care from the Eastern Finnish catchment population from 2005 to 2015 (flowchart in Fig. 2, Table 1). Of the 769 aSAH patients, 145 (19%) had died within 14 days of admission (Fig. 2, Table 1), a time period at neurointensive care considered acceptable for organ donation.

### Reconstruction of the clinical lifelines of the study population

The clinical lifelines of the 769 aSAH patients have been constructed using their clinical data from the Kuopio database and the national clinical registries until death or the last follow-up. For the 145 aSAH patients, dead within 14 days of admission, data in the National Organ Transplantation Center was reviewed for actual organ donations.

### Literature review

PubMed search in January 2018 for English articles in humans between 1995 and 2018 with the search words: (subarachnoid hemorrhage) and ((brain death) and (donor* or donation* or transplantation*) gave 43 hits. We reviewed these articles and their reference lists, and identified four relevant cohorts [7, 12, 15, 17].

### Definition of potential organ donors (PODs) in the present study

In 2010, an international expert panel (neurology, neurotraumatology, intensive care medicine, transplantation medicine, intensive care ethics, and organ procurement management) suggested criteria for intensive care to identify a patient with a reasonable probability to become brain dead (imminent brain death) after acute brain catastrophe [3]. Two validated coma scales (Glasgow Coma Scale and the FOUR Score), brain stem reflexes, and respiration were used to define imminent brain death (Table 2). The algorithm defined an actual pool of potential organ donors (PODs), with the exclusion of advanced age and medical contraindications.

**Table 2:** Definition of potential organ donors (PODs) in a previous multi-disciplinary consensus, as compared to the present study of aSAH patients

de Groot YJ, et al. 2010 [3]: Imminent brain death: point of departure for potential heart-beating organ donor recognition	
* Known origin of catastrophic brain damage * Condition considered irreversible * Ventilated patient * GCS 3 * Absence of *3 out of 6* brain stem reflexes * Exclusion criteria: age; medical contraindications	
The present study	
* Catastrophic brain damage after aSAH * Condition considered irreversible * Ventilated patient * GCS 3 * Absence of *2 out of 6* brain stem reflexes * No exclusion by age or medical conditions on admission	

Brain stem reflexes: pupillary reactivity to light, corneal reflex, oculocephalic response, oculovestibular responses, gag reflex, and cough reflex

*aSAH* aneurysmal subarachnoid hemorrhage, *GCS* Glasgow Coma Scale

In the present study, we analyzed in retrospect PODs among the 769 aSAH patients until death within 14 days after acute admission to neurointensive care. The POD criteria above were used, with two exceptions: absence of only two brain stem reflexes, no strict exclusion criteria for advanced age or medical conditions on admission (Table 2).

### Statistical methods

The categorical variables were expressed in proportions and the continuous variables in medians, quartiles, and ranges. Groups were compared using the Pearson *χ*2 test, the Mann–Whitney *U* test, or the independent sample *t* test, when appropriate. Independent risk factors for becoming a POD after aSAH were examined using the logistic regression analysis enter method. *P* values < 0.05 were considered significant. SPSS 22 statistical software was used for the analyses (SPSS, Inc., Chicago, IL).

### Ethical aspects

The Research Ethics Committee of the Kuopio University Hospital approved the study. Data fusion from the national registries was performed with approval from the Ministry of Social Affairs and Health of Finland. Kuopio Neurosurgery Intracranial Aneurysm Study Group has received consent from all patients recorded in the database. A separate written consent for the national register data fusion was not required by the Ministry of Social Affairs and Health.

## Results

### Study population

Between 2005 and 2015, a total of 769 aSAH patients (Fig. 2, Table 1) had been acutely admitted to KUH Neurointensive Care from a defined catchment population (Fig. 1), and 145 (19%) of them had died within 14 days of admission (flowchart in Fig. 2). Table 2 presents the distribution of their clinical variables.

### Potential organ donors

Of the 145 aSAH patients who died within 14 days, 62 (43%) had died of cardiopulmonary causes: their median age was 69 years (range 40–86) and 34 (55%) had drug-treated hypertension (Table 2). We retrospectively identified 83 (57%) potential organ donors (PODs) (Table 2), using the POD criteria in Table 1 in “Materials and Methods.” Their median age was 56 (range 30–85) and 29 (34%) had hypertension. Reflecting their moribund condition on admission, 57 (69%) had not had occlusive therapy of the ruptured aneurysm, as against 93 (12%) of all 769 aSAH patients (Table 2). Severe condition on admission (Hunt and Hess grade IV or V) was independently associated with the eventual POD status (Table 3).

**Table 3:** Independent factors associating in multivariate analysis with potential organ donor (POD) status (*n* = 83) among the 769 consecutive aneurysmal subarachnoid hemorrhage (aSAH) patients acutely admitted to the Neurointensive Care of Kuopio University Hospital from its Eastern Finnish catchment population from 2005 to 2015

Variable	OR	*P* value	CI (95%)
Hunt and Hess grade IV	6	0.04	1.1–34
Hunt and Hess grade V (extension to pain)	20	0.01	3.3–117
No occlusion of aneurysm due to moribund condition	25	< 0.01	8.1–79

### Causes for non-donorship

Only 49 of the 83 PODs had become actual donors (Table 2), a donor conversion rate (DCR) of 59% in retrospect.

The causes for non-donorship among the 34 PODs were 15/34 (44%) refusals of consent; 18/34 (53%) patients with medical unsuitability for donation; and 1/34 (3%) violation of the protocol and failure to recognize POD (Fig. 2).

### Presumed consent and refusals of consent

Finland had implemented the national presumed consent (opt-out) within the study period in the end of 2010. In 2005–2010, there were 11 refusals by near relatives with DCR 52% (29/56), but only three in 2011–2015 with DCR 74% (20/27). There was one patient who had denied own organ donation during life.

### Decompressive craniectomy

Of the 769 aSAH patients, 46 (6%) had had decompressive craniectomy (DC), an option through the study period (Table 1). Small sample size did not allow the evaluation of the impact of DC on relative mortality rates.

## Discussion

### Essential results of the present study

We analyzed 769 consecutive acute aSAH patients in a prospectively collected database, admitted between 2005 and 2015 to the neurointensive care unit of a university hospital, solely serving a defined catchment population, also using their data from the national transplantation registry and other clinical registries. In our population-based practice, we started in 2004 to admit virtually all aSAH patients to acute neurointensive care, also as PODS, almost regardless of the age (oldest 87 years), previous medical conditions, or dismal prognosis for the survival. In retrospect, we identified 83 (57%) potential organ donors (PODs) among the 145 aSAH patients who died within 14 days of admission. Severe condition on admission (Hunt and Hess grades IV or V) independently associated with the eventual POD status (Table 3). Of the 83 PODs, only 49 had become actual donors, due to 15 refusals of consent and 18 medical conditions unsuitable for donation. Finland had implemented national presumed consent (opt-out) within the study period in the end of 2010. There were 11 refusals in 2005–2010 with the donor conversion rate (DCR) of 52% and only three in 2011–2015 with DCR of 74%.

Severe clinical condition on admission for acute aSAH in defined catchment populations associates with significant mortality in neurointensive care. In our previous study, the mortality of 1657 aSAH patients (1980–2007) was 22% at 30 days [10]. The independent risk factors were Hunt and Hess grade V (OR 43) or IV (OR 10), age over 65 years (OR 3.9), aneurysm size over 25 mm (OR 3.7), and IVH (OR 1.8). In the present study, 71% of the grade V patients and 26% of the grade IV patients died within 14 days, a time period at neurointensive care considered acceptable for organ donation in our practice. On the other hand, some of the grade V patients may recover with a favorable outcome [4, 13].

In a Dutch university hospital, 179 (38%) of the 473 SAH patients (1999–2003) had died during neurointensive care. Of the 132 PODs, 35 had been treated until determination of the brain death. With nine refusals, 26/132 (DCR 20%) became organ donors [12]. In a German university hospital, 71 (18%) of the 395 aSAH patients (2011–2016) admitted to the intensive care unit had died [15]. Of the 36 PODs, 23 (DCR 64%) became actual organ donors. In 13 patients, the consent had been declined by a written will of the patient or by their closest relatives.

### The limitations and strengths of our study

The limitation of our study is that the POD status was evaluated retrospectively, albeit from prospectively collected data in the database. The strengths derive from the Finnish health care system. Finland is divided into mutually exclusive catchment areas among the 5 university hospitals, allowing disease cohorts that are unselected and minimally biased. The Kuopio Intracranial Aneurysm Patient and Family Database reliably reflects aSAH in the Eastern Finnish population and allows reconstruction of the clinical lifelines of aSAH patients, also using data from the national clinical registries. In Finland, all organ removals and transplantations are performed and archived by the Transplantation Center of the Helsinki University Hospital.

### Finnish national policies to enhance donor action

Finland implemented national presumed consent (opt-out) in the end of 2010, applying to brain-dead patients over 18 years of age and with previous full capacity to make binding amendments to their rights. The near relatives are informed of the brain death and asked whether the brain-dead patient had during life expressed refusal of own organ donation in case of own brain death. The national goal is that 70% of adults would express their opinion on organ donation by 2018. The Finnish National Archive for Health Information (www.kanta.fi/en/) provides every citizen a possibility to enter own opinion to an online platform, accessible at all times to health care professionals. The near relatives do not have a legal right of vetoing organ donation, but strict refusals are respected in clinical practice. There were 11 refusals in 2005–2010 with the DCR of 52% and only three in 2011–2015 with the DCR of 74%. The numbers are too small to argue that the presumed consent as such would have increased DCR, and increased awareness of the donor action in neuroacutology and neurointensive care, but our study suggests that it may play a role.

Furthermore, a recent Finnish guideline recommends that moribund brain catastrophe patients should be admitted to intensive care as PODs, regardless of dismal prognosis for the survival, along a dedicated organ donation program for the catchment population. The impact of these national policies remains to be verified in large population-based cohorts. Finally, the association of decompressive craniectomy and DCR in intensive care of aSAH patients should be studied in large collaborative cohorts [1]. The essential question is whether decompressive craniectomy improves the survival of aSAH patients rather than merely increases the time to eventual death.

### Conclusions

Nearly 20% of all aSAH patients acutely admitted to neurointensive care from a defined catchment population died within 14 days, almost half from cardiopulmonary causes at a median age of 69 years. Of all aSAH patients, 11% were considered as potential organ donors (PODs). Donor conversion rate (DCR) was increased from 52 to 74% after the national presumed consent (opt-out). Implicitly, DCR among aSAH patients could be increased by admitting them to the intensive care regardless of dismal prognosis for the survival, along a dedicated organ donation program for the catchment population.

## Abbreviations

aSAH: aneurysmal subarachnoid hemorrhage
DCR: donor conversion rate
GCS: Glasgow Coma Scale
ICH: intracerebral hematoma
ICP: intracranial pressure
IVH: intraventricular hematoma
POD: potential organ donor
TBI: traumatic brain injury.
